# History, versatility and future prospects of oscillatory carbonylation reactions of alkynes

**DOI:** 10.1039/d1ra03810a

**Published:** 2021-07-12

**Authors:** Katarina Novakovic, Lev Bruk, Oleg Temkin

**Affiliations:** School of Engineering, Newcastle University Newcastle upon Tyne NE1 7RU UK katarina.novakovic@ncl.ac.uk; MIREA-Russian Technological University (Lomonosov Institute of Fine Chemical Technologies) Moscow 119571 Russia

## Abstract

The paper looks back at three decades of oscillatory carbonylation reactions, summarises core findings and shares perspectives, with particular emphasis on applications. Oscillatory carbonylation reactions of alkynes display remarkable versatility in terms of substrates, catalysts and solvents. Furthermore, in addition to oscillations in pH and redox potential, these organic chemical oscillators can yield oscillations in turbidity and release heat from the reaction in a pulsatile manner. Recent research developments shift attention from small molecule substrates (*e.g.* phenylacetylene) and small molecule catalysts (*e.g.* palladium(ii) iodide), to oscillatory carbonylation reactions using polymeric substrates (*e.g.* PEGylated alkynes) and polymeric catalysts (*e.g.* imine-functionalized chitosan-palladium) and use of these polymeric building blocks to develop oscillatory (pulsatile) materials fit for pulsatile drug release and other applications.

## Abstract 2

The *BT-GN reactions* are versatile chemical oscillators, oscillating in pH, redox potential and turbidity, and releasing heat in a pulsatile manner. The reaction system has a potential to be coupled with intelligent materials and yield applications.

## Graphical abstract

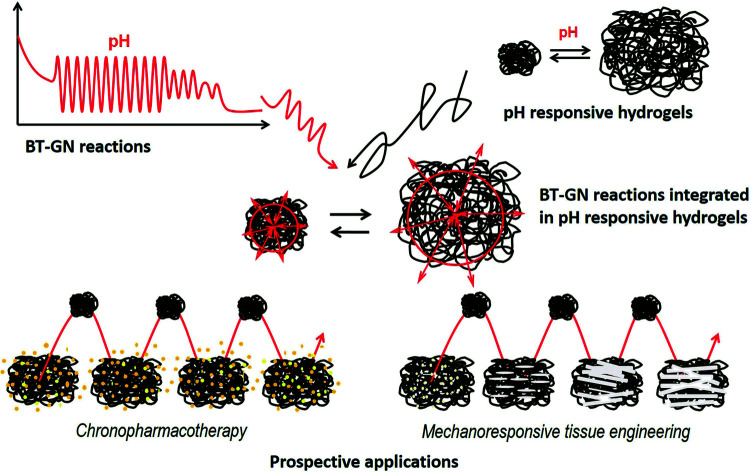

## Introduction

Discovered by Bruk, Temkin and their co-workers at the Lomonosov Moscow State Institute of Fine Chemical Technology in the 1990s,^1^ and further studied by Gorodskii and co-workers,^2^ oscillatory carbonylation reactions remained confined to the Russian Federation for well over a decade. During this time, for the worldwide research community pursuing oscillatory chemical reactions and related phenomena, oscillatory carbonylation reactions remained more of a curiosity than a recognized chemical oscillator. It was not until the 2000s, at the suggestion of Prof. Steve Scott (Leeds University) and keen interest from Novakovic *et al.*^3^ that this chemical oscillator came to Great Britain and in particular Newcastle University, where it is studied to this day. Named *BT-GN reactions*, after the core contributors (Bruk, Temkin, Gorodsky and Novakovic), these oscillatory organic reactions of alkynes have the potential to serve as the example of organic oscillators we can grow a new family of oscillatory reactions and oscillatory materials from.

This article is dedicated to the memory of Dr Sergey Gorodsky, who passed away in March 2019. Sergey was one of only a few scientists who studied oscillatory carbonylation reactions of alkynes. Unsurprisingly, this mesmerizing chemical oscillator became his passion.

## The discovery

**Fig. 1:**
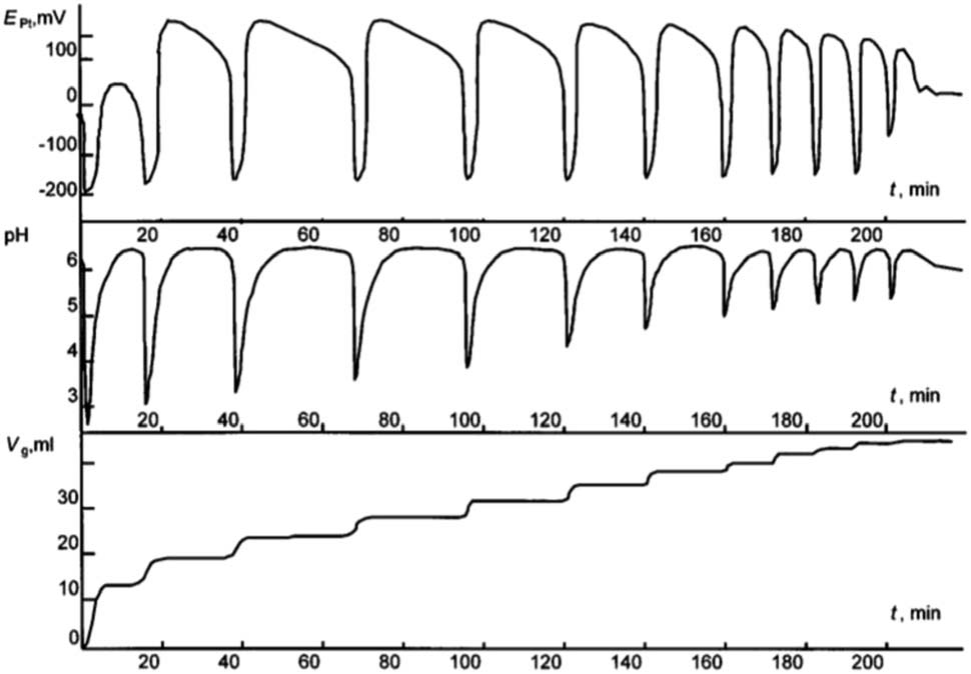
Oscillations of the platinum electrode potential (*E*_Pt_), pH, and volume of the gas mixture CO–O_2_ (*V*_g_, ml) consumed in the course of reaction at 40 °C. Initial concentrations: [PdI_2_] = 0.01 mol L^−1^, [KI] = 0.4 mol L^−1^, [PA] = 0.1 mol L^−1^, [NaOAc] = 0.0024 mol L^−1^; solvent used was methanol, and water (2 mmol) was added together with methanol to maintain the constant concentration of water at the initial level (approx. 0.2 mol L^−1^). This figure has been adapted from ref. 1 with permission from ACS Publications, copyright 1997 American Chemical Society.

In 1999, while working on the carbonylation of alkynes, Lev Bruk and his student Alexander Malashkevich, started studying the process of oxidative carbonylation of phenylacetylene (PA) in methanol solutions of palladium(ii) iodide complexes and made an important discovery. It was clearly established for the first time that the synthesis of complex organic molecules (diesters of phenylmaleic and phenylfumaric acids and dimethoxyphenyllactone) from simple substrates (PA, CO, CH_3_OH and O_2_) proceeds in the course of a catalytic reaction in a self-oscillating mode.^1^ Process was run in a well stirred and closed batch reactor at 40 °C under atmospheric pressure of 1 : 1 CO–O_2_ mixture, with the reactor connected to the biuret containing 1 : 1 CO–O_2_ mixture under atmospheric pressure. Malashkevich noticed that gas (CO + O_2_) absorption takes place intermittently in portions, with breaks (aligned with oscillation periods) reaching 27 minutes under certain conditions, and process gradually ending after 3.3 hours (Fig. 1).

Previously, such an unusual phenomenon in organometallic catalysis was observed in the work of G. M. Shulyakovsky, A. N. Nyrkova and O. Temkin in 1983–1985 when studying the reaction of carbalkoxylation of acetylene in solutions of palladium(ii) bromide complexes in the *n*-butanol–dimethylsulfoxide (DMSO) system with the addition of PPh_3_ ligand.^4^ Starting from acetylene, CO and butanol in the absence of oxygen, instead of obtaining butyl acrylate, diesters of maleic and fumaric acids and an oligomer containing carbonyl-, vinyl- and (CH_2_)_*n*_-groups are formed, and the process proceeded in an oscillatory mode. Oscillations were denoted in the rate of absorption of gas C_2_H_2_ + CO and the potential of a platinum electrode. CO, C_2_H_2_, CO_2_ and Me_2_S were found in the composition of the gas leaving the reactor in a stationary mode. In this system DMSO acts as the oxidant to form dimethylsulfide, CO_2_, and diesters. The self-oscillations also occurred in the absence of the PPh_3_ ligand.^4^ At the time, it was assumed that the oscillatory regime in this system is a rare exception, mainly due to the formation and destruction of new dimethylsulfide bromide complexes of palladium. However, following the findings reported by Malashkevich *et al.*,^1^ interest rose and studies of *BT-GN reactions* with the involvement of S. N. Gorodsky began.^2,5–7^ In 2012, Temkin summarises their findings thus far in his book,^8^ where he both elaborates on a mechanism and presents a kinetic simulation of this reaction proceeding in a self-oscillating mode.

## BT-GN reactions – early findings

**Fig. 2:**
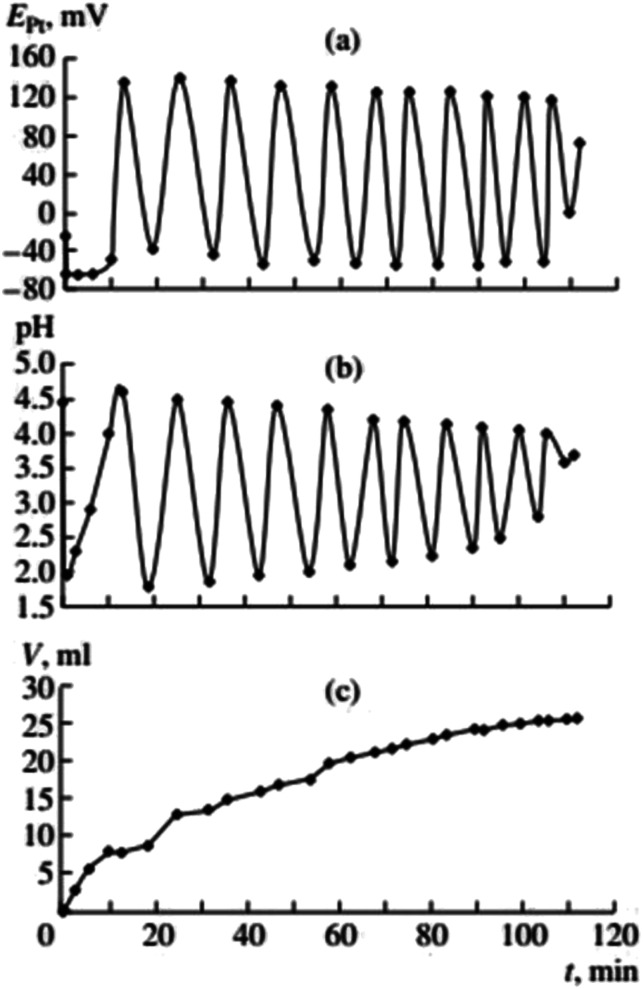
Changes in *E*_Pt_, pH, and the volume of consumed gases in the oxidative carbonylation of phenylacetylene (daylight, 700 rpm, [KI]_0_ = 0.4 mol L^−1^; [PdI_2_]_0_ = 0.01 mol L^−1^; [PA]_0_ = 0.1 mol L^−1^; [CO]_0_ : [O_2_]_0_ = 3 : 2). This figure has been reprinted from ref. 2 with permission from Springer Nature, copyright 2001 Original Russian Text Copyright.

In the early studies of carbonylation of PA, in the acid-free PdI_2_–KI–MeOH system at mild temperature (Fig. 2, ref. 2), the following observations were made.

After the dissolution of palladium iodide and potassium iodide in MeOH at 40 °C, pH values in the solution were 7–8, and the potential difference of platinum and silver chloride electrode (*E*_Pt_) in the range of 50–100 mV. After the air in the reactor was substituted with the CO/O_2_ mixture (without stirring of the solution), the pH fell to 5–6, and *E*_Pt_ to ∼0 mV. After stirring commenced and PA was added, three stages of the process were denoted (Fig. 2):

(1) *The first stage* (2–3 min) associated with the absorption of CO and O_2_, is characterised by a sharp decrease in the value of *E*_Pt_ (from between 0 and −20 mV, to between −70 and −100 mV), drop in pH (from 4.5–5 to 2.0–2.5) and the decrease in concentration of PA by 20–30% of the initial value.

(2) During *the second stage*, starting 2–3 min after the PA was added, and ending 8–12 min later, *E*_Pt_ remains constant or slowly increases, pH increases to 3–4, and the absorption of the gas mixture continues. At this stage changes in *E*_Pt_ and pH are not synchronised.

(3) At the end of the second stage, the catalytic system is developed and the reaction enters a self-oscillatory mode. *Third stage*, starting at 10–15 min from the beginning of stirring is denoted by a sharp increase in *E*_Pt_ (by 150–200 mV), the pH value reaching 4–5, and the system going into a mode of developed self-oscillations, accompanied by synchronous changes in *E*_Pt_ and pH, as well as stepwise gas absorption during periods within each oscillation corresponding to the decrease in values of *E*_Pt_ and pH.

**Fig. 3:**
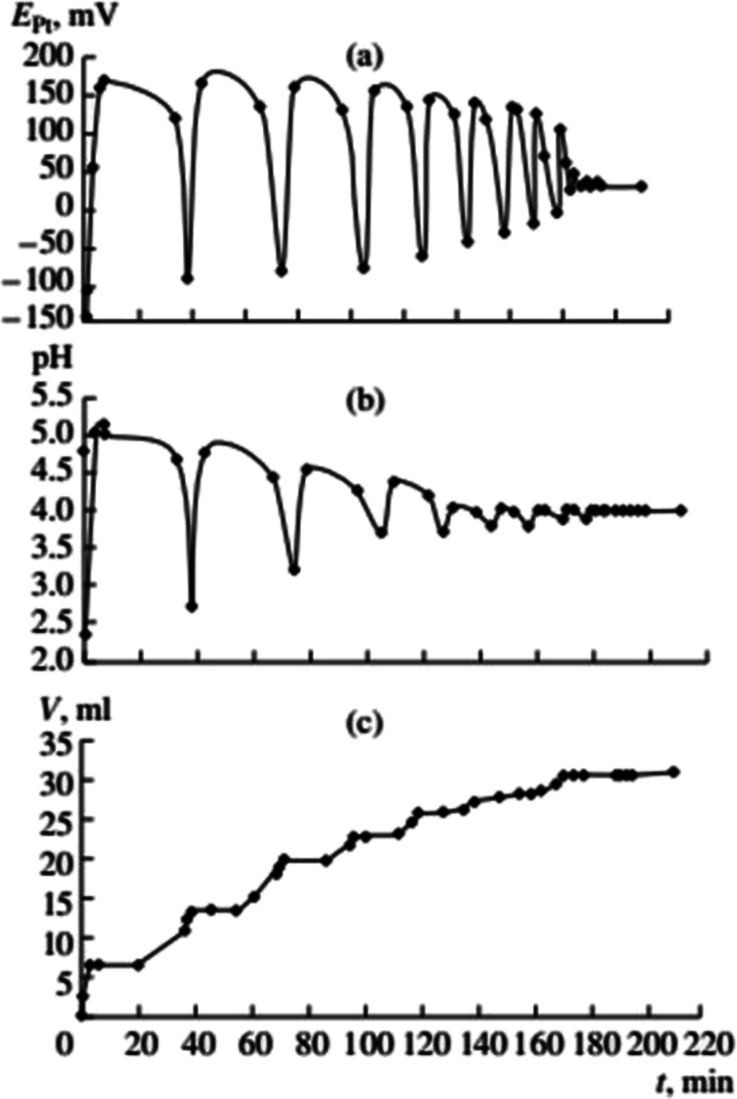
Changes in *E*_Pt_, pH, and the volume of consumed gases in the oxidative carbonylation of dimethyl ethynyl carbinol (DMEC) (700 rpm, [KI]_0_ = 0.4 mol L^−1^; [PdI_2_]_0_ = 0.01 mol L^−1^; [dimethylethynyl carbinol]_0_ = 0.1 mol L^−1^; [CO]_0_ : [O_2_]_0_ = 3 : 2). This figure has been reprinted from ref. 2 with permission from Springer Nature, copyright 2001 Original Russian Text Copyright.

Gorodskii *et al.*^2,5–7^ showed that oxidative carbonylation in self-oscillatory mode occurs for other alkynes in addition to PA (methylacetylene, 1-nonyne, dimethyl ethynyl carbinol (Fig. 3), propargyl alcohol). It was demonstrated that the nature of the alkyne, the composition of the catalytic solution, the ratio of the reaction gases and the intensity of mixing, affect the rate of the process and the characteristics of the oscillations (period and amplitude) in pH and *E*_Pt_.

For the interpretation of the periodic changes in the pH and the potential of the platinum electrode observed during the carbonylation of alkynes, it was important to relate these electrochemical characteristics to the state of the components of the catalytic system. While it was obvious that the pH values are associated with the changes in the acidity of the system, the potential difference between platinum and silver chloride (reference) electrodes (*E*_Pt_) is more challenging to correlate due to the presence of several possible oxidation states of palladium. As recognised, the potential of a platinum electrode is determined by the ratio of the activities of metal compounds (in this case, palladium) in the highest and the lowest oxidation states present in solution. Since the presence of palladium(iv) complexes in a noticeable amount at room temperature in the absence of strong oxidants is unlikely, and palladium(0) also cannot accumulate in the reaction system, because it is not sufficiently stabilized by either carbon monoxide or iodine anions,^9^ it was assumed that the main forms of palladium present in the catalytic solution and affecting the potential of the platinum electrode are palladium(ii) and palladium(i).^2,5^

Starting from the studies of conjugated processes of carbonylation of alkynes in the solution of palladium complexes, hypotheses about the activity of Pd(i) complexes in the carbonylation of alkynes and alcohols were formulated, substantiated, and translated to the nonlinear nature of the activity of Pd(i) complexes in the *BT-GN reaction* mechanism.^9–12^ The proposed mechanism of carbonylation included nonlinear stages of the formation of active complexes with elements of autocatalysis, stages of the transformation of alkynes into products of oxidative carbonylation, and nonlinear stages of the disappearance of active centres.^2,5^

Drop in the *E*_Pt_ and pH upon purging the reactor with a mixture of CO and O_2_ (*the first stage*), is associated with the oxidation of CO to CO_2_ with participation of trace water in methanol ([H_2_O]_0_ = 0.05 M) (1).

1 CO + H_2_O + PdI_2_ → CO_2_ + HPdI + HI

The conversion of PdI_2_ (into hydride complex occurs even faster after the introduction of PA into the reactor). One of the processes during the second stage is the reaction (2):

2 PdI_2_ + CO + PhC≡CH + MeOH → products + HPdI + HI

Similarly, a formation of palladium catalysts in homogeneous solution was observed in several processes of carbonylation of alkynes proceeding in a stationary mode.^10,11^ According to the well-established mechanism of oxidative carbonylation of alkynes,^9–12^ the products (mainly dimethylester of maleic and fumaric acids) are formed as a result of the redox decomposition of carboxyl and acyl σ-organometallic palladium complexes, leading to the formation of hydride complex (I, II): [Pd](COOH) → [Pd]H + CO_2_, and [Pd](COR) + MeOH → [Pd]H + MeOCOR.

Iodide palladium complexes do not form stable carbonyls^9^ and therefore stabilization of hydride iodide complexes of palladium is unlikely with carbon monoxide. Interaction with alkynes and oxidizing agents is likely to lead to the disappearance of the H–Pd bond. Therefore, the accumulation of significant amounts of HPdX particles in solution in the absence of stabilizing ligands, for example phosphines,^13^ is unlikely. The most probable pathway for the transformation of hydride complexes is their decomposition to palladium(0) complexes in solution (step 3) or oxidation by various oxidants present in the system.

3 HPdI ↔ HI + Pd(0)

Sometimes, when the process at the second stage is faster due to the high concentration of PA, palladium, or water, the precipitation of metallic palladium is observed. Oxidizing agents in the original *BT-GN reaction* system are O_2_ and PdI_2_ ([PdI_4_]^2−^).^1^ At the sufficiently high concentrations of palladium(ii), a nonlinear elementary step (4), leads to the formation of Pd(i) complex (probably Pd_2_I_4_^2−^):

4 HPdI + PdI_2_ → Pd_2_I_2_ + HI

**Fig. 4:**
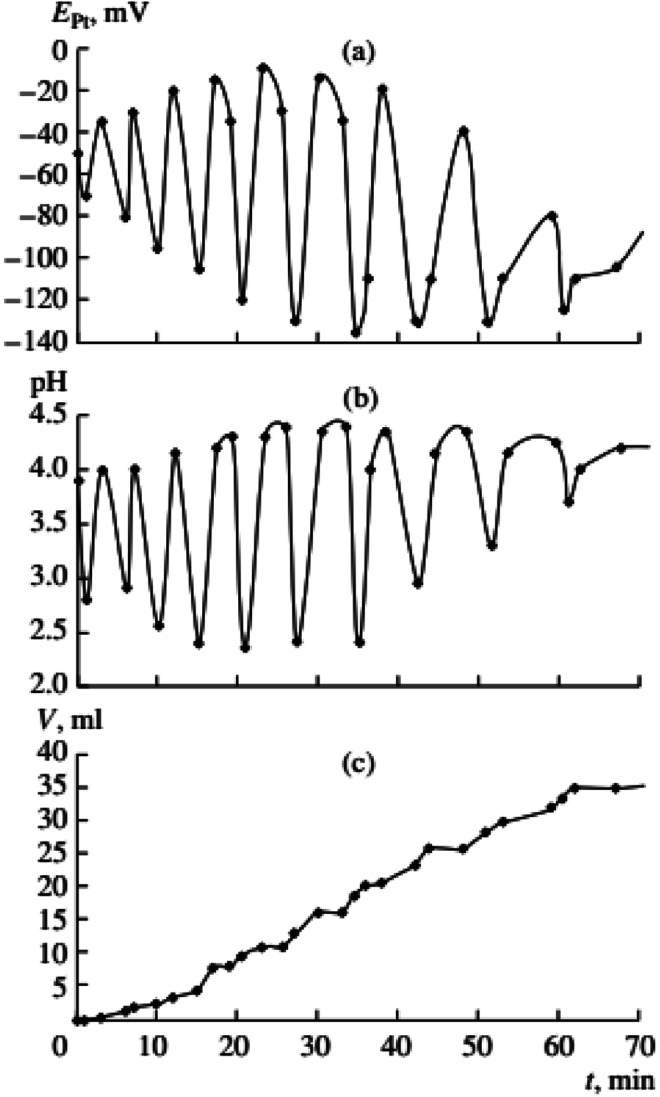
Changes in *E*_Pt_, pH, and the volume of consumed gases in the oxidative carbonylation of phenylacetylene with the potassium complex of Pd(i) (700 rpm, [KI]_0_ = 0.4 mol L^−1^; [Pd(i)]_0_ = 0.02 mol L^−1^; [PA]_0_ = 0.1 mol L^−1^; [CO]_0_ : [O_2_]_0_ = 3 : 2). This figure has been reprinted from ref. 2 with permission from Springer Nature, copyright 2001 Original Russian Text Copyright.

**Fig. 5:**
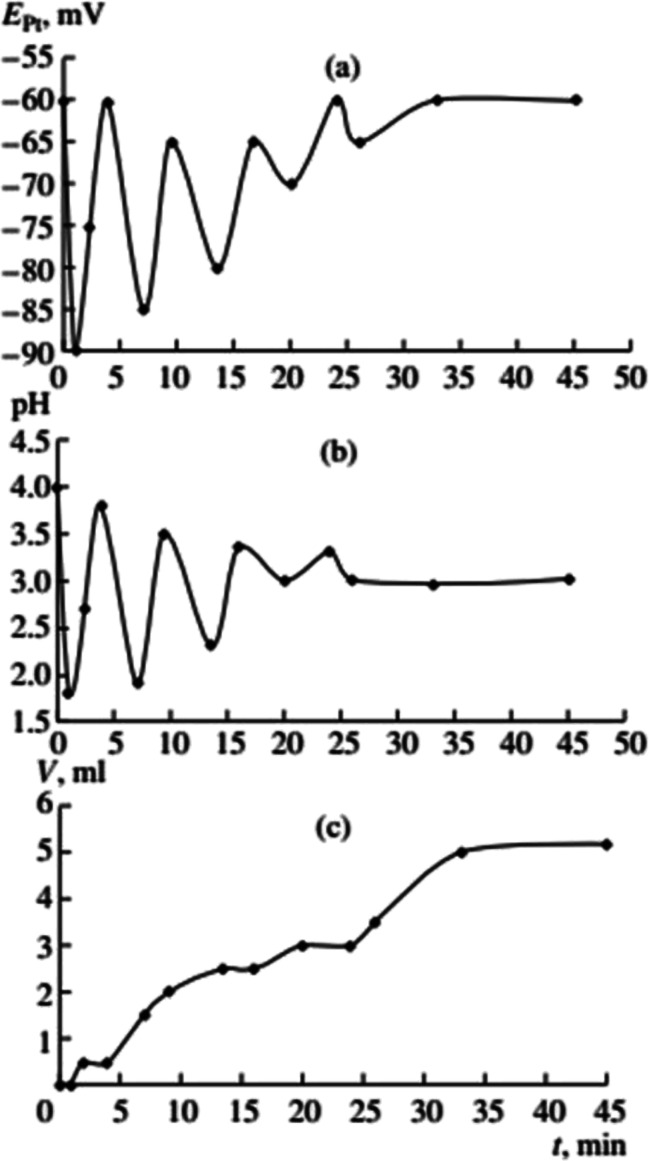
Changes in *E*_Pt_, pH, and the volume of consumed gases in the oxidative carbonylation of phenylacetylene with the caesium complex of Pd(i) (700 rpm, [KI]_0_ = 0.4 mol L^−1^; [Pd(i)]_0_ = 0.02 mol L^−1^; [PA]_0_ = 0.1 mol L^−1^; [CO]_0_ : [O_2_]_0_ = 3 : 2). This figure has been reprinted from ref. 2 with permission from Springer Nature, copyright 2001 Original Russian Text Copyright.

Previous data suggests palladium(i) compounds are active in the carbonylation of alkynes.^9–12,14^ To test this hypothesis palladium(i) complexes, KPd_4_I_5_ and CsPd_4_I_5_·12H_2_O, were synthesized following the method given in ref. 14. When PdI_2_ in the initial solution was replaced by an equivalent amount of the Pd(i) complex, self-oscillations in *E*_Pt_ and pH began immediately after the introduction of PA (without first and second stages I and II),^2^ (Fig. 4 and 5). These results support the hypothesis of higher activity iodide complexes Pd(i) in the oxidative carbonylation of PA in comparison with Pd(ii) compounds (5).

5 Pd_2_I_2_ + 2CO + PhC≡CH + 2MeOH → MeOOC(Ph)=CHCOOMe + 2HPdI

Reactions (4) and (5) taken together form the autocatalytic reduction of Pd(ii) with HPdI as auto-catalyst. It is in the reduced state (judging by the values of *E*_Pt_) that the catalyst exhibits maximum activity (see Fig. 1 and 2). The reaction (5) for dimethylesters as products contains a number of elementary steps including a set of intermediates: [Pd]OR, [Pd]COOR, [Pd](CH=C(Ph)COOR) and [Pd](COCH=C(Ph)COOR).^8–12^

The beginning of the second stage of the process is associated with a significant decrease in the PdI_2_ concentration and an increase in the concentrations of hydride complexes and Pd(i) compounds. This can lead to an acceleration of the oxidation of hydrides by oxygen or those oxidants that can be formed from it (steps (6)–(8)):

6 HPdI + O_2_ ↔ IPdOOH

7 IPdOOH + HI → PdI_2_ + H_2_O_2_

8 IPdOOH + HPdI → Pd_2_I_2_ + H_2_O_2_

The occurrence of these reactions can explain the increase in the pH and *E*_Pt_ values during the third stage. Oxidation of hydride complexes with molecular oxygen by the mechanism (6) and (7) was previously reported,^15^ but the formation of hydrogen peroxide is not experimentally confirmed. Oxidation of hydrogen iodide with oxygen leads to the formation of one more oxidant – molecular iodine, which is able to oxidize hydride complexes, palladium(0)^12^ and Pd_2_I_2_.

9 2HI + O_2_ → I_2_ + H_2_O_2_

10 HPdI + I_2_ → PdI_2_ + HI

11 Pd + I_2_ → PdI_2_

12 Pd_2_I_2_ + I_2_ → 2PdI_2_

A study devoted to the oxidation of palladium(i) with iodine showed that the accumulation of oxidants leads to the oxidation of palladium(i) compounds to palladium(ii) and the increase in the pH and *E*_Pt_ values, and likely is responsible for the complex dynamics of the self-oscillatory process.^10^

The steps listed above were used as a basis for putting forward hypothetical mechanisms which can describe the dynamics of the self-oscillatory process. A difficulty was encountered in the estimation of the kinetic constants, since the usual algorithms based on the minimization of functions, including the sum of the squares of the differences between the calculated and the experimentally measured concentrations, were ineffective in this case. In this regard, the values of some constants of the Oregonator model were used as an initial approximation for key nonlinear steps.^16^ By enumerating the chemical steps and the values of the kinetic constants, the simplest mechanism was found that made it possible to obtain a *qualitative* agreement between the model and the experimental results.

A simplified version of the mechanism may be represented by reaction (1), (4), (5), (9), (11) and (13), which is the sum of steps (3), (6) and (7):

13 2HPdI + O_2_ → PdI_2_ + Pd(0) + H_2_O_2_

**Fig. 6:**
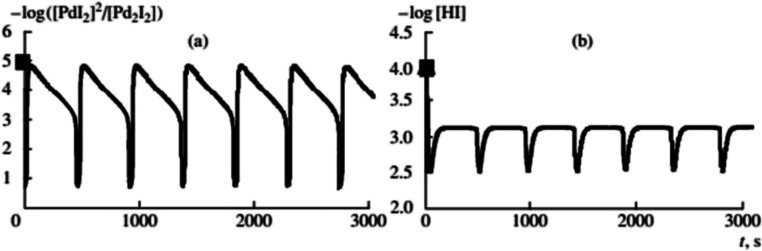
Results of the mathematical modelling of the oscillatory oxidative carbonylation of phenylacetylene ([Pd(i)]_0_ = 0.01 mol L^−1^, [PA]_0_ = 0.1 mol L^−1^). This figure has been reprinted from ref. 2 with permission from Springer Nature, copyright 2001 Original Russian Text Copyright.

The system of differential equations, formulated in accordance with the above chemical equations, together with a selected set of kinetic constants, made it possible to qualitatively describe the experimentally observed trends in the rate of this carbonylation process as well as periodic changes in the redox potential and *pH of the* solution, Fig. 6.^2^

## *BT-GN reactions* – calorimetry studies and selective product formation

**Fig. 7:**
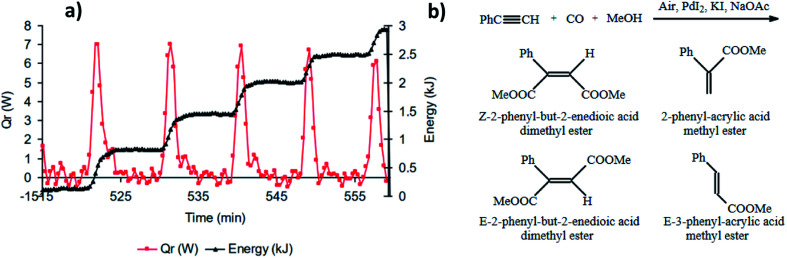
(a) Synchronised oscillations in pH and heat release. First methanol (400 mL) and PdI_2_ (2.03 g, 5.60 mmol) were stirred at 550 rpm and the temperature was set at 40 °C. Following KI (37.39 g, 225 mmol) and NaOAc (114 mg, 1.40 mmol) in 50 mL of methanol were added, and after a further 20 min, simultaneous purging of the system with CO and air (50 mL min^−1^ each) commenced. Thirty minutes after purging started, PA (6.2 mL, 56.5 mmol) was added; (b) reaction products. This figure has been adapted from ref. 3 with permission from Elsevier, copyright 2006 Elsevier B.V.

Interest in the oscillatory nature of the oxidative carbonylation of alkynes was taken by Novakovic *et al.* who, in addition to experimentally confirming pH oscillations in the original *BT-GN reaction* reported by Malashevich *et al.*,^1^ also recorded synchronised oscillations in the reaction heat output which were exothermic with no corresponding endotherm and in phase with a pH fall (Fig. 7a).^3^ By performing experiments in a reaction calorimeter with precise control of temperature and CO/air flow rates (reaction was purged with gasses, rather than gasses being provided *via* the headspace only) it was noted that total energy released during oscillations follows a staircase function (with a maximum of 600 J per oscillation) indicating a stepwise product formation; which is in agreement with the findings from a modelling study conducted by Gorodskii *et al.*^6^ which suggested a stepwise consumption of PA during the course of the reaction in oscillatory mode.

**Fig. 8:**
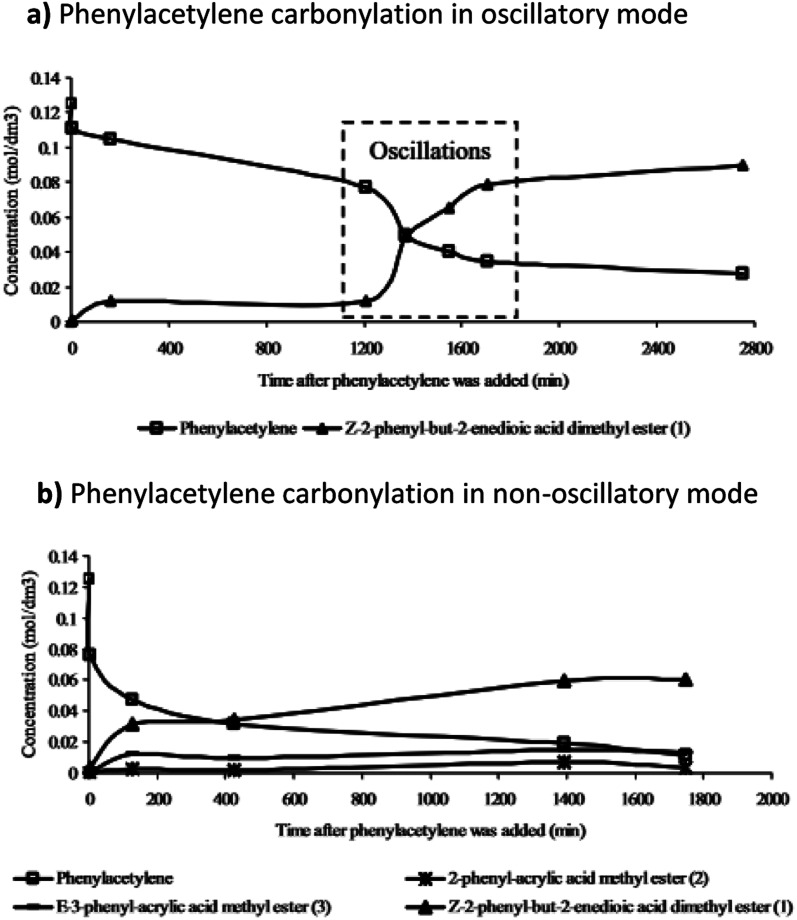
The product formation in (a) oscillatory and (b) non-oscillatory mode (related to PdI_2_ granularity) and the effect oscillations had on product selectivity and dynamics of product formation. Reproduced from ref. 17 by permission of the PCCP Owner Societies.

Further studies of the product formation in the oscillatory carbonylation reaction of PA revealed that occurrence of pH oscillations influences product selectivity.^17^ As shown in Fig. 8, when operating in an oscillatory mode at 40 °C, a high selectivity towards the formation of *Z*-2-phenyl-but-2-enedioic acid dimethylester was recorded. Furthermore, product formation was suppressed until oscillations occurred when a steep increase in reaction rate was observed. Rate of product formation, as expected, correlated with energy release.^17^

Difference in product distribution between non-oscillatory and oscillatory carbonylation of alkynes has been previously postulated by Gordskii *et al.*^6^ who concluded that in carbolylation reaction of alkynes multiple mechanistic pathways can take place. By broadening studies of *BT-GN reactions* employing PA as substrate to a wider temperature range (0–40 °C) it was demonstrated that in oscillatory mode reaction temperature affects product selectivity. Under otherwise identical conditions, at 40 °C the major product was *Z*-2-phenyl-but-2-enedioic acid dimethylester (as previously reported in ref. 17) while at 0 °C the major product was 5,5-dimethoxy-3-phenyl-2(5*H*)-furanone.^18^ The study noted that the conversion of PA to products recorded at 0–20 °C follows kinetic laws (conversions increase as the temperature increases) which becomes less apparent at 30 °C and is not followed at 40 °C, suggesting thermodynamic reaction control. Parker and Novakovic observed that the ratio of 5,5-dimethoxy-3-phenyl-2(5*H*)-furanone and *Z*-2-phenyl-but-2-enedioic acid dimethylester remains constant during the reaction and decreases with increasing temperature. This suggests the activation energies of the two pathways leading to formation of these two products differ, resulting in reaction temperature dictating which process will dominate, kinetically or thermodynamically controlled. For all temperatures studied (0, 10, 20, 30 and 40 °C), the stepwise release of energy occurred during the reaction, in correlation with the pH fall within each oscillation, providing further evidence of stepwise conversion of reactant to products, in full agreement with previous reports.^18,19^ Importantly, all temperatures studied showcased the benefits of a slow product formation in this *BT-GN reaction* and its ability to proceed in self-oscillatory mode for days and weeks with no further addition of either substrate (PA) or catalyst (palladium(ii) iodide). Experimentally Parker and Novakovic^20^ showed that synchronous with pH oscillations, oscillations in turbidity take place, strongly indicating oscillatory cycling between soluble and insoluble catalytic species (postulated to be Pd^2+^ and Pd^0^ respectively) in autocatalytic mode; supporting the suggestion of nonlinear and autocatalytic steps of generation and termination of active centres put forward by Temkin and Bruk.^21^ Further experimental studies of *BT-GN reaction* employing phenylacetylene demonstrated feasibility of self-oscillatory mode when the water concentration in the system is increased (0–30 vol%, mixed with methanol).^22^ Addition of water transformed oscillations in pH from regular oscillations to more stepwise behaviour. By establishing the relationship between the recorded pH and HI concentration it was found that increased water concentration suppresses HI formation and hence slows the autocatalysis in the reaction. These findings show a dilution with water (as well as potential for other solvents) as a significant mean in tailoring amplitude, period and duration of oscillations, but also as a way to reduce overall toxicity of this system.

## BT-GN reactions in multifunctional polymeric materials

**Fig. 9:**
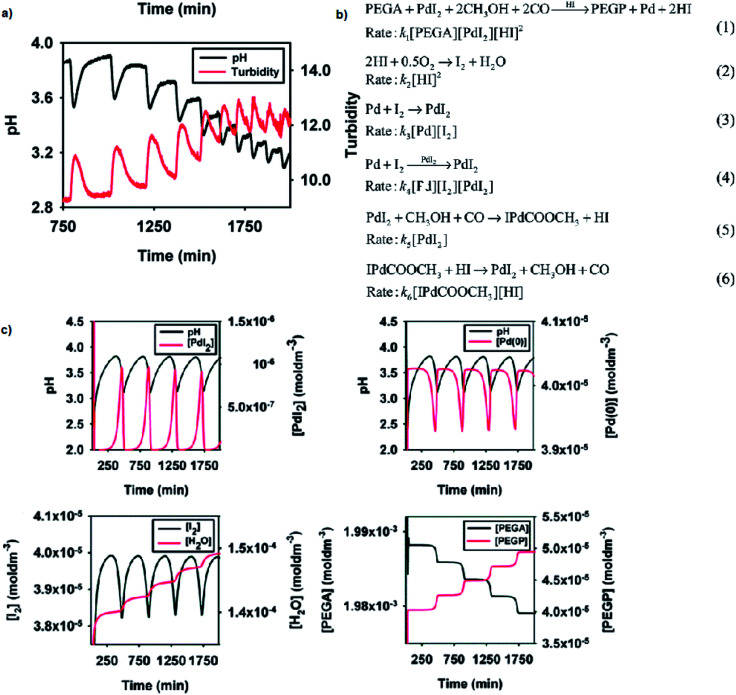
(a) Oscillations in pH and solution turbidity recorded in palladium-catalysed oxidative carbonylation of pegylated monoalkyne (5000 Da) in methanol. Initial conditions: [PdI_2_] = 4.05 × 10^−5^ M; [PEGA] = 2.03 × 10^−3^ M; [KI] = 2.28 × 10^−3^ M; CO flow = 15 mL min^−1^; air flow = 15 mL min^−1^. (b) Reaction network proposed to account for the experimentally recorded oscillations with PEGA. Concentrations of CH_3_OH, O_2_ and CO are in excess, therefore considered constant and were not included in the reaction rates. This assumption is experimentally confirmed and is valid for this particular system. (c) Example simulation study in BatchCAD using the reaction network given in Fig. 9b. Initial conditions: [PdI_2_] = 4.05 × 10^−5^ M; [PEGA] = 2.03 × 10^−3^ M. Rate constants (min^−1^): *k*_1_ = 6 × 10^12^, *k*_2_ = 2 × 10^3^, *k*_3_ = 1 × 10^−7^, *k*_4_ = 2 × 10^7^, *k*_5_ = 3 × 10^−4^, *k*_6_ = 1 × 10^2^. Results are validated using both adaptive Euler and adaptive Runge–Kutta integrators. Adapted from ref. 26.

Reported versatility of substrates, catalysts and solvents yielding self-oscillatory mode in the *BT-GN* processes^1–3,6,17–25^ reached a new height with the studies reporting self-oscillatory carbonylation reactions employing polymeric substrates (pegylated mono and dialkyne),^26–28^ and polymeric catalysts (Pd-polyacrylate, proline-functionalised chitosan-Pd, chitosan-palladium catalysts^27,28^), and expanding the choice of solvents to a broad range of aliphatic alcohol (methanol, ethanol, 1-propanol, 1-butanol and 1-hexanol), including less toxic ethanol.^27^ Example experimental study employing pegylated monoalkyne (PEGA), core model sufficient to reproduce experimental observations and simulation study is presented in Fig. 9a–c, respectively. As can be noted from the reaction scheme (Fig. 9b) two autocatalytic reactions have been postulated (eqn (1) and (4)). The reaction in eqn (1) is assumed to be autocatalytic in HI to account for the sharp rise in hydrogen ion concentration as well as product (PEGP) formation and catalyst reduction. The reaction presented in eqn (2) accounts for the formation of iodine, which is subsequently responsible for the regeneration of the palladium catalyst (eqn (3) and (4)). Eqn (2) also accounts for hydrogen ion consumption. Catalyst recycling is postulated to proceed in both a non-catalytic and autocatalytic manner, eqn (3) and (4) respectively. Autocatalysis supports the recorded oscillatory behaviour of solution turbidity whilst eqn (3) allows for catalyst regeneration in the event of full consumption of PdI_2_. Eqn (5), although experimentally recorded as a slow and reversible process,^29^ is included as it accounts for the initial formation of hydrogen ions required to initiate the reaction given in eqn (1). Reversibility is acknowledged by eqn (6). Due to the excess of CH_3_OH, O_2_ and CO their concentrations were considered constant^19,29,30^ and were not included in the reaction rates.

**Fig. 10:**
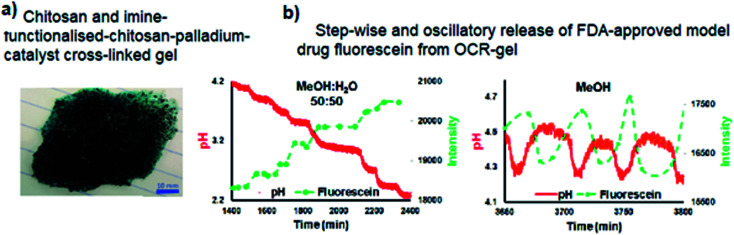
(a) Chitosan-imine-PdCl_2_ macrogel – top view; (b) pH-controlled release of fluorescein as a function of time in methanol : water and methanol systems. Inverse photoluminescence intensity is presented as closed symbols. The connecting lines are only as a guide for the eye and do not represent data. Reproduced from ref. 31 with permission from The Royal Society of Chemistry.

Transitioning *BT-GN reactions* from solution chemistry to polymer science opened new opportunities and led to the recent reports of pulsatile hydrogels capable of drug release in a stepwise manner (Fig. 10a and b).^31^ In their experimental study, Isakova and Novakovic demonstrated a proof-of-concept self-oscillatory chitosan macrogel, employing the palladium-catalysed oxidative carbonylation reaction as the driving force of its oscillations. The reported hydrogel (Fig. 10a) was composed of highly biocompatible components and an imine-functionalised chitosan-palladium catalyst with zero leaching rates. During the synthesis macrogels were loaded with FDA-approved model drug fluorescein and following when used in place of catalyst in *BT-GN reaction*, shown to rhythmically release model drug fluorescein. The step-wise release pattern corresponded to the step-wise dynamics of pH decrease in methanol : water, while in pure methanol, the changes in pH had an oscillatory mode, accompanied by mirrored oscillations in fluorescein concentration (Fig. 10b).

## Future prospects of *BT-GN reactions*

Combining oscillatory chemical reactions and smart hydrogels in a single system has long been seen as a way forward to producing novel functional oscillatory materials capable of numerous applications where autonomous change in material conformation is required. Following ground-breaking proof of concept and BZ-gels fabricated by Yoshida *et al.*,^32^ expectations rose. However, the leap to meaningful application is yet to take place. What *BT-GN reactions* showed so far indicates the potential these systems have to transition to a relevant application. PEGylated alkyne substrates not only showcase the versatility of oscillatory carbonylation reactions but also open the opportunity to reduce toxicity of substrate and envision way forward of incorporating polymeric substrate within the smart hydrogel matrix, *e.g.* as interpenetrating polymer network or *via* grafting process. Equally among numerous polymeric catalysts demonstrated viable in *BT-GN reactions*, non-leaching Pd-chitosan stands out as it introduces proven biocompatible hydrogel and effectively enhances biocompatibility of the whole system. Furthermore, replacing methanol with ethanol and ethanol/water mixtures is seen as another way of reducing the overall toxicity of the system. Lastly, as this is carbonylation reaction, CO is required in low concentrations (mM). While studies can be focused towards replacing CO with less toxic counterparts, it is worth noting the endogenous production (12 mL day^−1^) and physiological functions of CO,^33^ blood CO levels and body CO stores^34^ that may serve as a trigger of oscillations in pulsatile hydrogels *in vivo*. Equally, advanced modelling studies of the system are needed to complement and support experimental efforts. Recently, the Kolar-Anic group used Stoichiometric Network Analysis to examine instabilities in oscillatory carbonylation of poly(ethylene glycol)methylether acetylene^35^ and also expand on previously proposed reaction network in order to obtain a more realistic reaction model.^36,37^ In the new model, the direct autocatalytic steps were replaced with autocatalytic loops and the expressions for reaction rates correspond to their stoichiometry in accordance to mass action kinetics. It is of paramount importance to continue studies of oscillatory chemical reactions and pursue the development of oscillatory materials for the multitude of prospective applications, for example as actuators, drug delivery systems, self-driven micro-robots and tissue engineering scaffolds. In that view, the *BT-GN reaction* family is well worthy of our attention both as a versatile chemical oscillator with a strong potential to achieve meaningful applications but also as a knowledge-bank that may lead to the discovery of new chemical oscillators.

## Conflicts of interest

There are no conflicts to declare.

## Supplementary Material
